# Comprehensive analysis of m6A-modified circRNAs in peritoneal metastasis of high grade serious carcinoma of ovary

**DOI:** 10.3389/fonc.2022.988578

**Published:** 2022-09-20

**Authors:** Lin Guo, Nini Xu, Daner Qiu, Xiaozhe Yang, Shasha Zhao, Hongxi Zhao

**Affiliations:** Department of Obstetrics and Gynecology, Tangdu Hospital, The Fourth Military Medical University, Xi`an, China

**Keywords:** high grade serious carcinoma of ovary peritoneal metastasis, circular RNA, N6-methyladenosine, m6A-modified circRNAs, HGSOC

## Abstract

**Purpose:**

High-grade serous ovarian cancer (HGSOC) remains the most lethal female cancer due to metastasis. CircRNAs are recently identified to be modified by N6-methyladenosine (m^6^A) in many cells. However, the significance of m^6^A-modified circular RNAs (circRNAs) has not been elucidated in HGSOC peritoneal metastasis. Here, we aimed to investigate the participation and potential functions of m^6^A-modified circRNAs in HGSCO peritoneal metastasis.

**Methods:**

Cancerous tissues were collected from the *in situ* and the peritoneal metastasis lesions of HGSCO patients. M^6^A-tagged circRNAs were identified by m^6^A-modified RNA immunoprecipitation sequencing (m^6^A-RIP-seq). Gene Ontology (GO) annotation and Kyoto Encyclopedia of Genes and Genomes (KEGG) pathway enrichment analyses were performed to predict the potential functions of the m^6^A-modified circRNAs.

**Results:**

For the m^6^A-modified circRNAs, 259 were upregulated and 227 were downregulated in the peritoneal metastasis than in the situ lesions of HGSCO patients. For the m^6^A peaks, 1541 were upregulated and 1293 were downregulated in the peritoneal metastasis than in the *in situ* lesions of HGSCO patients. For the differential expressed circRNAs, 1911(19.6%) were upregulated and 2883(29.6%) were downregulated in the peritoneal metastasis than in the *in situ* lesions of HGSCO patients. The upregulated m^6^A-modified circRNAs were associated with the HIF-1 signaling. The downregulated m^6^A-modified circRNAs were associated with the MAPK signaling.

**Conclusions:**

This work firstly identified the transcriptome-wide map of m^6^A-modified circRNAs in peritoneal metastasis of HGSCO. Our findings provided novel evidences about the participation of m^6^A-modified circRNAs *via* HIF-1 and MAPK signaling and a new insight in molecular target of HGSCO peritoneal metastasis.

## Introduction

Ovarian cancer (OC) is the 7^th^ most commonly diagnosed gynecologic malignancy worldwide, accounting for 2.5% of all female cancers (1, 2). In 2020, there were about 313,959 new cases and 207,252 deaths of OC worldwide in the world (3, 4). OC consists of subtypes with distinct biological and molecular properties, with epithelial ovarian cancer accounting for more than 95% and non-epithelial ovarian cancer accounting for 5%. OC is a heterogeneous disease that is histologically classified into 5 major subtypes: high-grade serous, low-grade serous, clear cell carcinoma, endometrioid carcinoma, and mucinous ovarian carcinoma (3, 4). High-grade serous ovarian cancer (HGSOC) is the most common histological subtype of OC and accounts for 67.5% of all OC subtypes (1, 2), and remains the most lethal female cancer with an estimated 21,410 new cases and 13,770 deaths expected in the United States in 2021 (5).

HGSOC is typically diagnosed at advanced stages owing to the deficiency of specific symptoms at early stages, which is characterized by the involvement of bilateral ovaries, aggressive behavior and low survival without effective molecular targeted therapy. To date, regardless of the continuous improvement and development of surgical and systematic management, the mortality of these patients has not been improved under the standard regimens with approximately 70% of patients having relapse and metastasis after treatment. Consequently, identifying the mechanisms that drive HGSOC metastasis will translate into clinical managements that would improve their outcomes (1, 5–8).

Currently, significant efforts have been made for personalized treatment to improve prognosis of HGSOC patients, including application of genomic technologies to identify the expression profile, copy-number changes, mutations, and epigenetic modifications of the genes. RNA sequencing (RNA-seq) is one of the key genomic technologies applied to reveal molecular pathogenesis in HGSOC (5–8).

Circular RNA (circRNA) is one type of noncoding RNA (ncRNAs) with a closed loop structure without 5′-3′polarity and poly A tail, which were firstly identified abundant in viruses by RNA-seq, and predominantly regulates the gene expressions at the transcriptional and post-transcriptional levels (9–12). Moreover, it has been reported that circRNAs are closely associated with HGSOC after circRNAs high-throughput sequencing (13).

Over 160 different chemical modifications in RNAs have been identified in all living organisms Among these RNA modifications, N6-methyladenosine (m^6^A), methylated at the N6 position of adenosine, is identified as the most universal epigenetic modification on ncRNAs in eukaryotes and affects the circRNA expressions (14–16). M^6^A modification is predominantly enriched near the stop codons, within the internal long exons and at the 3′untranslated regions (14, 17). The m^6^A methylation can be catalyzed by the methyltransferases (“writers”) such as METTL3/14/16, removed by demethylases (“erasers”) FTO or ALKBH5, and interacts with the m^6^A binding proteins (“readers”), such as YTHDF1/2/3 and IGF2BP1/2/3 (14, 18–20). The circRNAs displayed cell-type-specific M^6^A-methylation patterns in many human cells (14, 18, 20). The m^6^A-modifed circRNAs are expected to promote protein translation in a cap-independent pattern (14, 21). However, the m^6^A-modification of circRNAs in HGSOC remains unknown.

Therefore, we hypothesized that m^6^A-modified circRNAs might be related to the HGSOC metastasis by regulating the genes/signaling pathways associated with HGSOC development. Here, we conducted a genome-wide screening of m^6^A-modified circRNAs in the tissues of HGSOC patients with peritoneal metastasis to identify the expression profiling of the m^6^A-modified circRNAs. This study may provide new therapeutic targets for HGSOC patients.

## Materials and methods

### Reagents

TRIzol Reagent was bought from Invitrogen-Gibco (Carlsbad, USA). NEBNext rRNA Depletion Kit and NEBNext Ultra II Directional RNA Library Prep Kit New England Biolabs, Inc. (Ipswich, USA).GenSeqTM m6A-MeRIP Kit was bought from GenSeq Inc. (Cyberjaya, Malaysia). Anti-N6-methyladenosine (m^6^A) antibody and Rnase R were purchased from Merck Millipore-Sigma-Aldrich (Beijing, China).

### Patients, informed consents and ethics statement

All study protocols were approved by the Ethics Committee of Tangdu Hospital, the Fourth Military Medical University and performed following the principles of the Helsinki Declaration. Each included patient provided a written informed consent. Cancerous tissues were collected from the *in situ* and the peritoneal metastasis lesions of 3 HGSCO patients during the surgery treatment, and were applied for the initial high throughput screening of m^6^A- methylated RNA immunoprecipitation sequencing (MeRIP-seq) (22, 23).

### RNA extraction and quality control

Total RNA (10 mg) was obtained from the cancerous tissues by using the TRIzol Reagent following the manufacturer’s protocols. The extracted RNAs were purified with Rnase R (RNR07250, Epicentre) digestion to remove linear transcripts.

NEBNext rRNA Depletion Kit was used to remove rRNAs from the total RNAs. The RNA concentration was measured at 260/280 nm (1.8 to 2.1) by using NanoDrop ND-2000. Total RNA quality was evaluated with the band intensity ratio of 18S/28S ribosomal after electrophoresis in an ethidium bromide-containing 1% agarose gel (22, 23).

### RNA-seq analysis of circRNAs

After quality filtering, paired-end reads were collected from Illumina Hiseq Sequence. The reads were aligned to the reference genome (UCSC RN5) with STAR software. The CIRI software was applied to detect and annotate circRNAs. Raw junction reads were normalized against per million reads mapped to the genome by using log2 scaled (14).

### Library preparation and MeRIP-Seq for maps of m^6^A-methylated RNAs

To immunoprecipitate the m^6^A-methylated RNAs, the anti-N6-methyladenosine (m^6^A) antibody was cultured with the fragmented total RNAs extracted from the cancerous tissues of HGSCO patients after rRNA depletion with the GenSeqTM m^6^A-MeRIP Kit following the manufacturer’s instructions. The bound RNAs were eluted from the protein A/G magnetic beads after incubation.

The input RNAs and m^6^A-immunoprecipitated (m^6^A-IP) RNAs were reverse-transcribed to cDNA for the construction of the RNA-seq library by using NEBNext Ultra II Directional RNA Library Prep Kit. The library quality was weighed by using a BioAnalyzer 2100 system (Agilent Technologies, Inc., Santa Clara, USA). Then the qualified m^6^A-IP and input samples were subjected to 150 bp paired-end deep sequencing on Illumina HiSeq sequencer to obtain the high-resolution reads of m^6^A-methylated RNAs.

The RNA-seq of circRNAs and the m^6^A RNA-Seq were carried out by the Cloud-seq Biotech Inc. (Shanghai, China).

### Bioinformatics analysis

Paired-end reads were collected from Illumina HiSeq sequencer with the quality control of Q30. After 3’ adaptor-trimming, the low-quality reads were removed with the cutadapt software (v1.9.3). First, the clean reads of input libraries were aligned to the reference genome (UCSC HG38) by using the STAR software. Based on the STAR alignment results, the circRNAs were identified by using the DCC software. Then, the clean reads of all libraries were aligned to the reference genome with the Hisat2 software (v2.0.4). The MACS software was applied to identify the methylated sites on circRNAs (peaks) and the diffReps was further used to identify the differentially methylated sites. The overlapping sites between the peaks, identified by both software based on the circRNA exons, were selected by the home-made scripts.

### GO and KEGG analysis

The comprehensive function annotations of the source genes of differentially m^6^A methylated circRNAs were achieved by Gene Ontology (GO), as well as Kyoto Encyclopedia of Genes and Genomes (KEGG) enrichment analyses based on the online software DAVID.

### Statistical analysis

Statistical analysis was performed with SPSS 25.0 (IBM, Chicago, USA) and GraphPad Prism software 7.0 (GraphPad Software, San Diego, USA). The single factor ANOVA was used for statistical analyses, *p* < 0.05 was considered statistically significant (24).

## Results

### Abundance of m*^6^A-*methylated circRNAs and circRNAs involve in peritoneal metastasis of HGSCO patients

**Figure 1:**
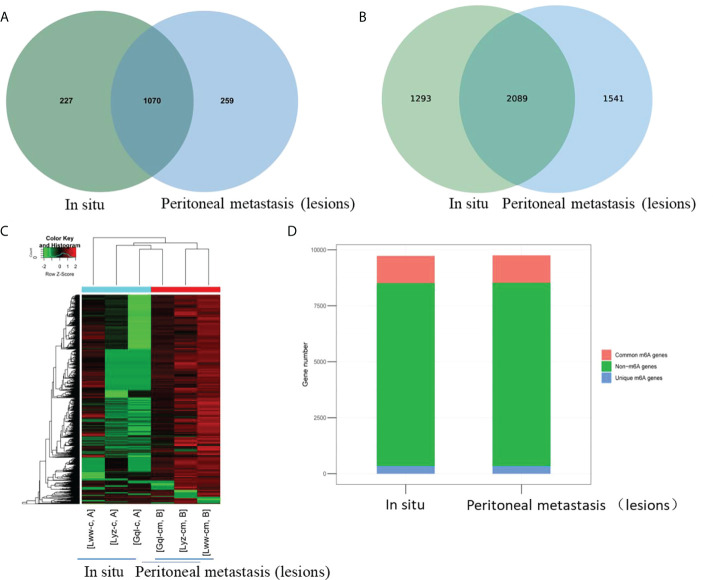
Overview of m^6^A-modified circRNAs of the *in situ* and the peritoneal metastasis lesions in the HGSCO patients. Vnn diagrams presenting the overlapped **(A)** m^6^A-modified circRNAs and **(B)** m^6^A peaks of the *in situ* and the peritoneal metastasis lesions in HGSCO patients. **(C)** Heatmap of the m^6^A-modified circRNAs. **(D)** Bar plots of the m^6^A peaks pear circRNAs.

A genome-wide profiling of m^6^A-modified circRNAs was performed in cancerous specimens of three biological replicates from the *in situ* lesions (n = 3) and the peritoneal metastasis lesions (n = 3) from HGSCO patients. Patient information is presented in **Table 1**. The MeRIP-Seq data had been submitted to gene expression omnibus (accession number, GSEXXX). As shown in the **Figures 1A, B**, the m^6^A abundance in the peritoneal metastasis lesions (1070 + 259 for the m^6^A-modified circRNAs and 2089 + 1541 for the m^6^A peaks) was found to be slightly increased than the *in situ* lesions (1070 + 227 for the m^6^A-modified circRNAs and 2089 + 1293 for the m^6^A peaks) of the HGSCO patients, which were further visualized by the Heatmap (**Figure 1C**) and Bar plots (**Figure 1D**).

**Table 1:** The demographic information of HGSCO patients.

Patient No.	Age	BMI	Pathological	Stage	Tissues for M^6^A RNA-Seq	
No.1	55	23.8	HGSCO	IIIC	*In situ*	Peritoneal metastasis
No.2	71	21.64	HGSCO	IIB	In situ	Peritoneal metastasis
No.3	43	22.66	HGSCO	IIB	In situ	Peritoneal metastasis

HGSCO, High grade serious carcinoma of ovary.

**Figure 2:**
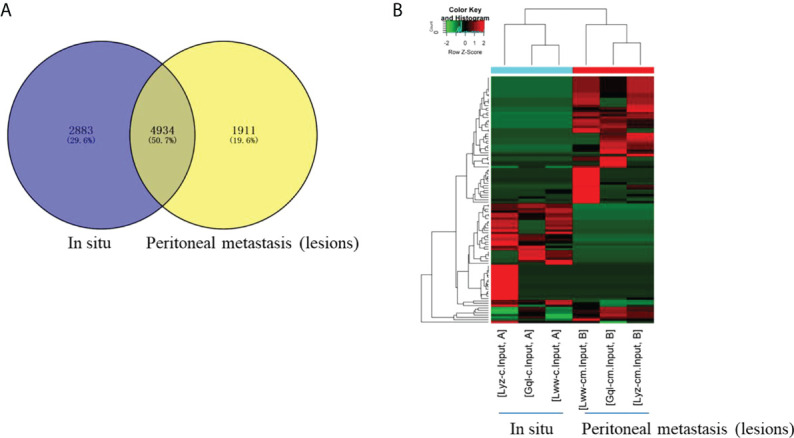
The differentially expressed circRNAs between the *in situ* and the peritoneal metastasis lesions of the HGSCO patients. **(A)** Vnn diagram and **(B)** Heatmap presenting the differential expressed circRNAs between the *in situ* and the peritoneal metastasis lesions of the HGSCO patients.

To explore whether m^6^A methylation would influence circRNA expression levels in HGSCO patients, RNA-seq analysis of circRNAs was further performed, and 4934 (50.7%) overlapping circRNAs were identified. CircRNA expression was decreased in the peritoneal metastasis lesions than in the situ lesions. These findings suggest that m^6^A may downregulate the circRNA expressions in peritoneal metastasis lesions of HGSCO patients (**Figure 2**).

### Distribution of circRNAs in the peritoneal metastasis lesions from HGSCO patients

**Figure 3:**
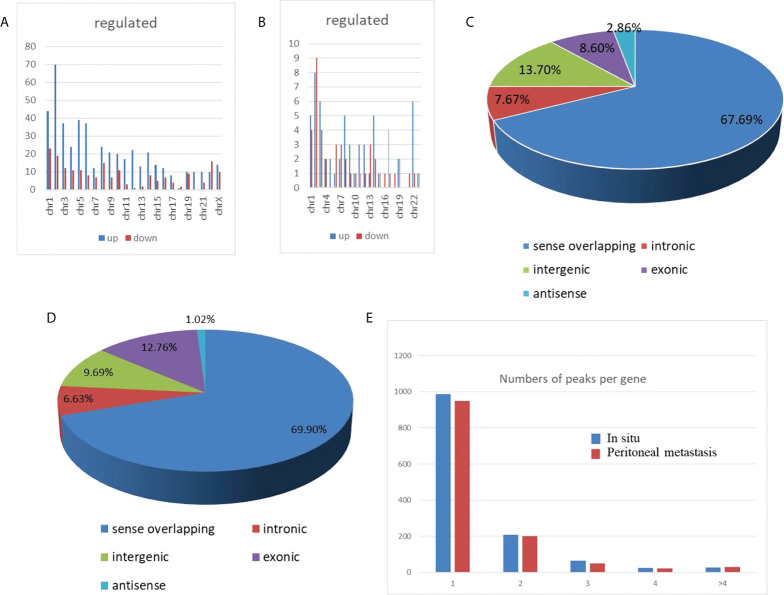
Chromosomal and genomic distributions of circRNAs. **(A)** Chromosomal distribution of differently m^6^A-modified circRNA peaks. **(B)** Chromosomal distribution of differently expressed circRNAs. **(C)** Genomic distribution of differently m^6^A-modified circRNA peaks. **(D)** Genomic distribution of differently expressed circRNAs. **(E)** The m^6^A peak numbers per circRNA.

To characterize the chromosomal and genomic distributions of all differently m^6^A-modified circRNA peaks and differentially expressed circRNAs of the peritoneal metastasis lesions than the *in situ* lesions from the HGSCO patients, the distributions of the down-regulated or upregulated m^6^A-modified circRNA peaks and the differentially expressed circRNAs on each chromosome was calculated and plotted. The upregulated m^6^A-modified circRNA peaks were mostly located in the chromosome 1, 2, 5 and 6 (**Figure 3A**), while the downregulated m^6^A-modified circRNA peaks were mostly located in chromosome 1, 2, 8 and 22 (**Figure 3A**). The upregulated differentially expressed circRNAs were mostly located in the chromosome 2, 3, and 22 (**Figure 3B**), while the downregulated m^6^A-modified circRNA peaks were mostly located in chromosome 1, 2 and 3 (**Figure 3B**). Genomic distribution of differently m^6^A-modified circRNA peaks (**Figure 3C**) and differently expressed circRNAs were mostly (over 65%) in the sense overlapping region (**Figure 3D**). Moreover, single m^6^A-modified circRNA peaks per gene was slightly decreased in the peritoneal metastasis lesions than the *in situ* lesions from the HGSCO patients, which was the predominant number of peaks per gene for both the peritoneal metastasis lesions and the *in situ* lesions from the HGSCO patients (**Figure 3E**), indicating that m^6^A methylation was abundant in circRNAs originated from single exons in both the peritoneal metastasis and the *in situ* lesions with a slight downregulation in the peritoneal metastasis lesion.

### Functional Annotation of the differently m*^6^A-modified circRNAs*

To explore the physiological and pathological significance of m^6^A modification in peritoneal metastasis of HGSCO patients, GO enrichment analysis and KEGG pathway analysis were conducted with the significantly changed m^6^A peaks. GO enrichment analysis was applied to predict the key functions of the target genes. Pathway analysis was accomplished to identify the significant pathways of the differential genes based on the KEGG.

**Figure 4:**
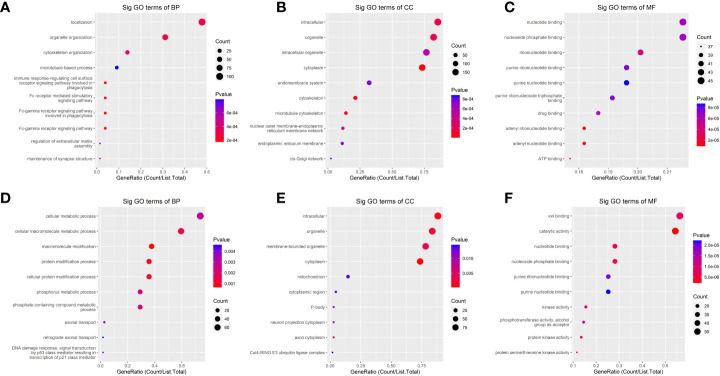
The Gene ontology (GO)-term enrichment analysis of significantly altered m^6^A-modified circRNAs between the *in situ* and the peritoneal metastasis lesions of the HGSCO patients. **(A–C)** upregulation of m^6^A-modified circRNAs. **(D–F)** downregulation of m^6^A-modified circRNAs.

In the GO analysis (**Figure 4**), the parent genes of circRNAs with upregulated m^6^A peaks were enriched in the localization and organelle organization, while downregulated m^6^A peaks were enriched in the cellular macromolecule metabolic process, the macromolecule modification, the protein modification process and the cellular protein modification process in the biological process (BP) analysis. The parent genes of circRNAs with upregulated m^6^A peaks were enriched in the intracellular, organelle and cytoplasm, while downregulated m^6^A peaks were enriched in the intracellular, organelle, cytoplasm and membrane-bounded organelle in the cellular component (CC) analysis. The parent genes of circRNAs with upregulated m^6^A peaks were enriched in the adenyl ribonucleotide binding and the adenyl nucleotide binding, while downregulated m^6^A peaks were enriched in the catalytic activity in the molecular functions (MF) analysis.

**Figure 5:**
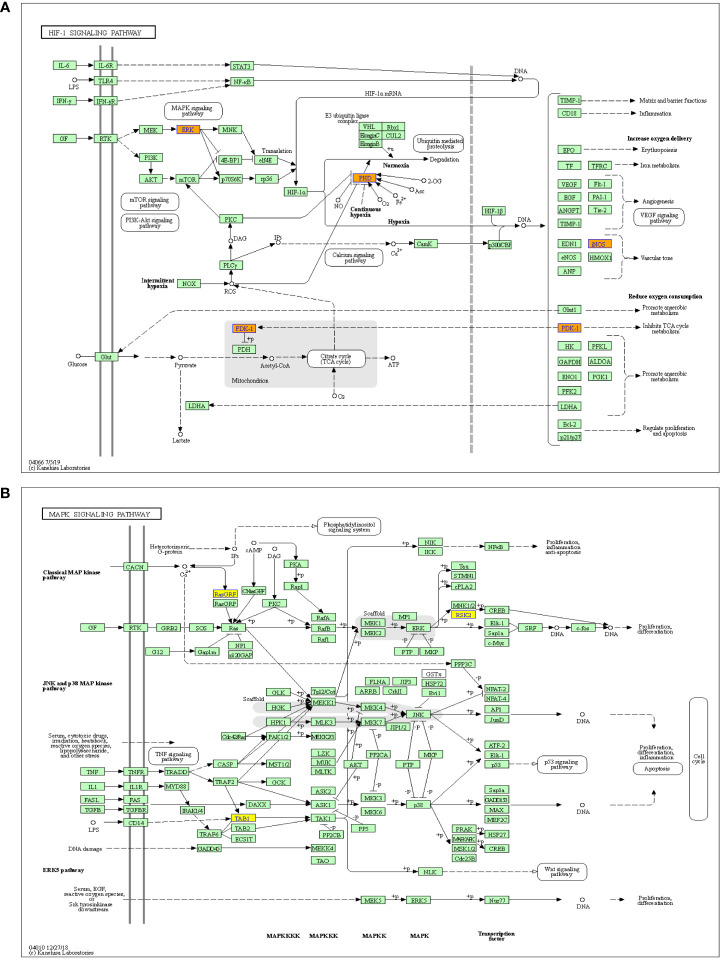
Pathway analysis of significantly altered m^6^A-modified circRNAs between the *in situ* and the peritoneal metastasis lesions of the HGSCO patients. **(A)** The up-regulated m^6^A-modified circRNAs involved in HIF-1 pathway. **(B)** The down-regulated m^6^A-modified circRNAs play important roles in MAPK signaling pathway.

Meanwhile, the HIF-1 and MAPK signaling pathways have been reported to be closely related with the cancer metastasis (25, 26), therefore, these two pathways were further analyzed. The results showed that the HIF-1 signaling pathway was associated with the upregulated m^6^A-modified circRNAs, while MAPK signaling pathway was associated with the downregulated m^6^A-modified circRNAs (**Figure 5**).

## Discussion

The overall survival rate of HGSOC has not been improved in the last three decades, and most patients develop recurrent disease within 3 years and died from the disease within 5 years. HGSOC is extremely regulated by epigenetic modifications (27). M^6^A plays an important role in numerous biological processes, which contributes to cancer development, cancer cell proliferation and cancer stem cell self-renewal (28, 29). The expression profile and potential function of circRNAs in different cancers have been identified, including HGSOC (13, 30, 31). As a new epitranscriptomic marker, m^6^A is recognized as a reversible and dynamic RNA modification in eukaryotes. Recently, m^6^A epigenetic modification in circRNA has attracted attention as a novel epigenetic event (22, 23, 32). However, the role of m^6^A epigenetic modification in circRNAs during HGSOC metastasis has not been characterized.

As far as we know, our current work was the first to explore the m^6^A-circRNA profile during peritoneal metastasis of HGSOC by using MeRIP-Seq. Our results support the concept of a dynamic characteristic of m^6^A epigenetic modification during peritoneal metastasis of HGSOC, which is associated with HGSOC development.

A genome-wide profile of m^6^A-modified circRNAs between the *in situ* and the peritoneal metastasis lesions were obtained by using MeRIP-seq. A total of 2089 m^6^A-modified circRNAs were shared in the peritoneal metastasis and the *in situ* lesions from HGSOC patients, whereas1293 of m^6^A-modified circRNAs were identified in the *in situ* lesions only but lacking in the peritoneal metastasis lesions, and 1541 m^6^A-modified circRNAs were identified in the peritoneal metastasis lesions only but lacking in the *in situ* lesions of the HGSOC patients.

Our results demonstrated that the expression of differentially regulated circRNAs was decreased in the peritoneal metastasis lesions than in the situ lesions by RNA sequencing, indicating that m^6^A may downregulate the circRNA expressions during the peritoneal metastasis of HGSCO.

Many questions have been raised with the findings of m^6^A-modified circRNA profile that need to be addressed in the future work, including the significance of m^6^A-modification on exons where the circRNAs originate. A recent work reported that m^6^A-modified circRNAs are more commonly encoded by single exons, which is likely to be longer than the multiexon circRNAs in human embryonic stem cells (18). Interestingly, this phenomenon has also been validated in our current work, which showed that single m^6^A-modified circRNA peaks per gene was predominant for both the peritoneal metastasis and the *in situ* lesions from the HGSCO patients, indicating that m^6^A methylation was abundant in circRNAs originated from single exons in both the peritoneal metastasis and the *in situ* lesions with a slight downregulation in the peritoneal metastasis lesion.

Studies have reported different rules which may control the biogenesis of m^6^A-modification in circRNAs compared with the mRNAs (18). Therefore, further studies are necessary to prove whether m^6^A-modified circRNAs present distinct modification patterns compared with the mRNAs between the peritoneal metastasis and the *in situ* lesions of the HGSOC patients.

A key function of circRNAs is to affect the biological roles of cells by regulating the target gene expressions. Thus, regulating the m^6^A-modification status in circRNAs may control the target gene expression of circRNAs and the cellular biological functions (33). CircRNAs mainly affect miRNAs and reduce their expression in regulating cytokine levels (34). Cancer is one of the leading causes of death worldwide, and the factors responsible for its progression need to be elucidated (35). CircRNAs could affect gene expression *via* targeting miRNAs to treat cancer (36). Therefore, in the present study, the GO and KEGG pathway enrichment analyses were carried out to explore the possible functions of changed m^6^A-modified circRNAs by combining analysis of differentially m^6^A-modified circRNAs and differentially expressed circRNAs associated with peritoneal metastasis of the HGSOC patients. We found that HIF-1 signaling was associated with the up-regulated m^6^A-modified circRNAs, while MAPK signaling was associated with the downregulated m^6^A-modified circRNAs, which will need to be further studied in the future.

In conclusion, the level of m^6^A abundance in total circRNAs was slightly increased in the peritoneal metastasis lesion from HGSOC than the *in situ* lesion, and m^6^A-modfication may downregulate the circRNA expressions during peritoneal metastasis of HGSCO. The bioinformatics analysis predicted the potential functions of m^6^A-modified circRNAs and the involved relevant signaling pathways, with HIF-1 pathway associated with the upregulated, while MAPK pathway associated with the downregulated m^6^A-modified circRNAs during peritoneal metastasis of HGSOC. Our findings provided evidences regarding the involvement of m^6^A-modifications in circRNAs of peritoneal metastasis of HGSOC, which might provide a new insight in the molecular target of HGSCO metastasis.

## Data availability statement

The datasets presented in this study can be found in online repositories. The names of the repository/repositories and accession number(s) can be found in the article/supplementary material.

## Ethics statement

All animal procedures were approved by the Research Ethics Committee of the Second Hospital of Hebei Medical University.

## Author contributions

Conceptualization, methodology, design, and writing: LG, NX, DQ, XY, and SZ. Supervision: HZ. All authors contributed to the article and approved the submitted version.

## Funding

This research has been supported by Health Research Funding of Shaanxi Province Grant (Number: 2022D063); Innovation Project of Tangdu Hospital (Grant Number: 2018QYTS009).

## Conflict of interest

The authors declare that the research was conducted in the absence of any commercial or financial relationships that could be construed as a potential conflict of interest.

## Publisher’s note

All claims expressed in this article are solely those of the authors and do not necessarily represent those of their affiliated organizations, or those of the publisher, the editors and the reviewers. Any product that may be evaluated in this article, or claim that may be made by its manufacturer, is not guaranteed or endorsed by the publisher.
